# Supplementary figures and images for: The impact of increasing expenditure on National Essential Public Health Services on the medical costs of hypertension in China: A difference-in-difference analysis
Source: PLoS One. 2022 Nov 28;17(11):e0278026. doi: 10.1371/journal.pone.0278026 (PMC9704679; doi:10.1371/journal.pone.0278026)

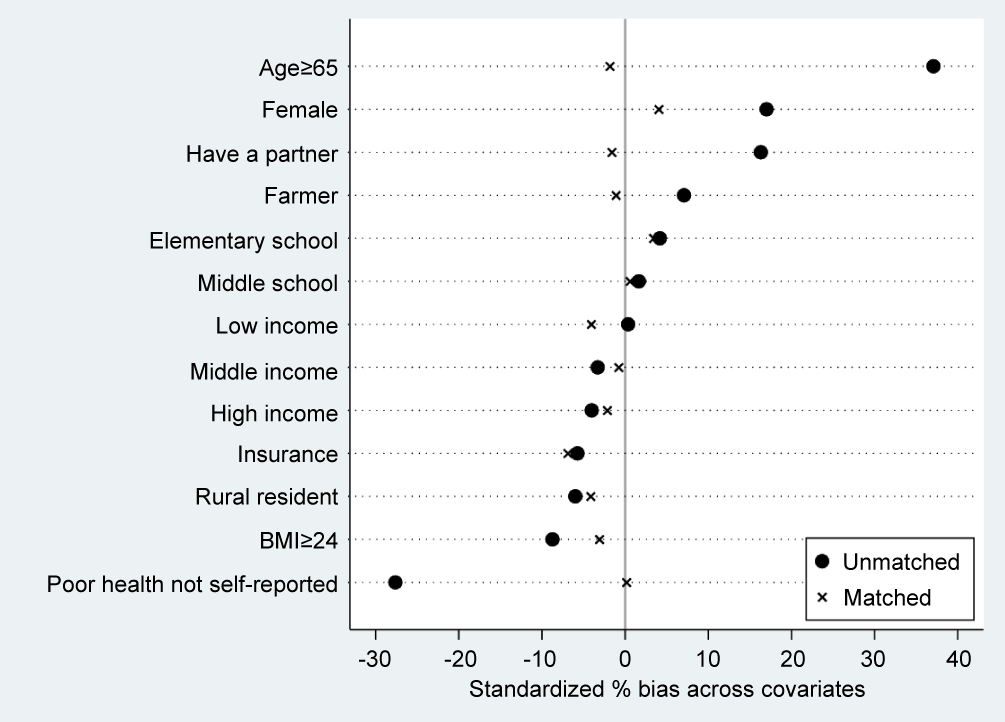

Supplement: S1 Fig — Illustrates the standardized bias across the covariates in the -10% to 10% range across the two groups’ propensity scores before and after matching, and the matching effect was satisfactory. (TIF) [file pone.0278026.s001.tif]

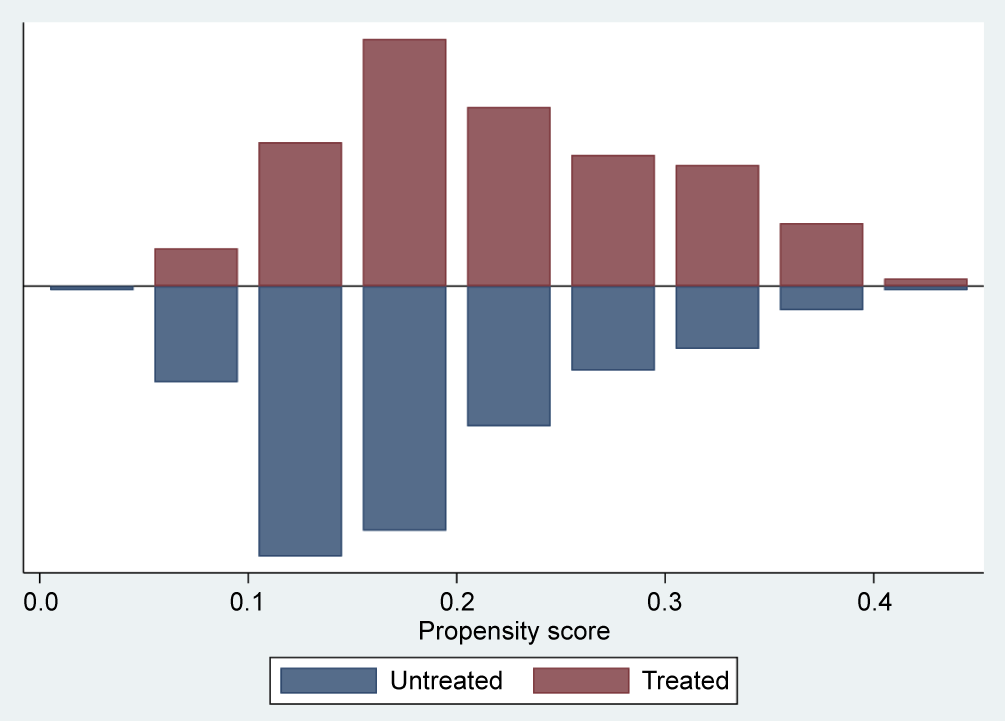

Supplement: S2 Fig — Illustrates the propensity score distribution across the two groups’ propensity scores before and after matching, and the matching effect was satisfactory. (TIF) [file pone.0278026.s002.tif]

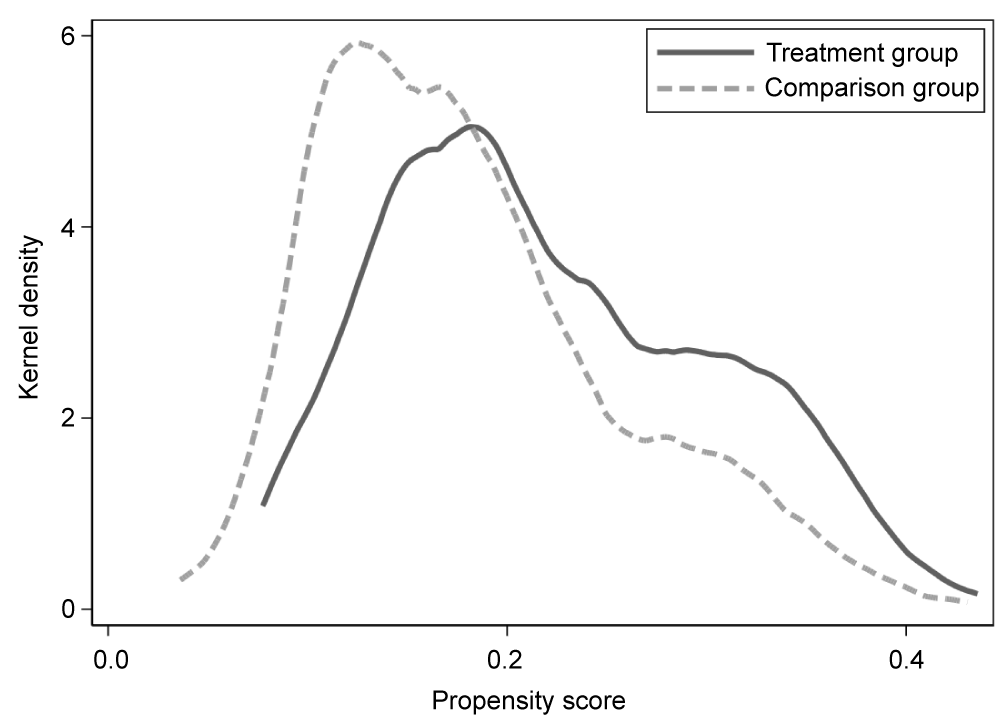

Supplement: S3 Fig — Illustrates the propensity kernel density across the two groups’ propensity scores before matching. (TIF) [file pone.0278026.s003.tif]

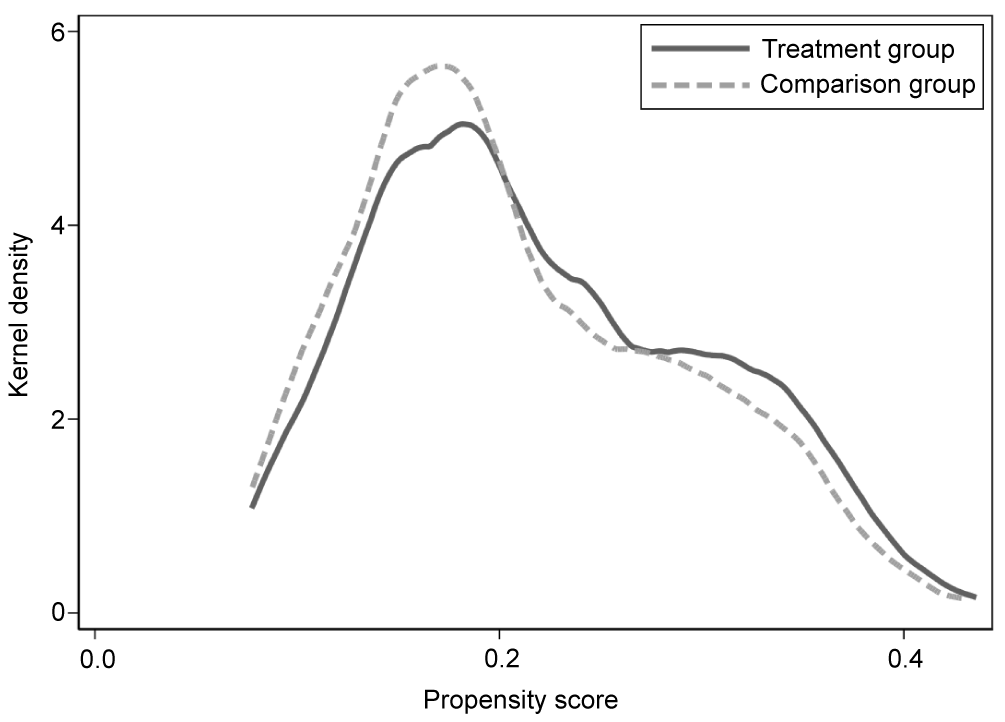

Supplement: S4 Fig — Illustrates the propensity kernel density across the two groups’ propensity scores after matching, and the matching effect was satisfactory. (TIF) [file pone.0278026.s004.tif]
