# Supplementary material for: The impact of increasing expenditure on National Essential Public Health Services on the medical costs of hypertension in China: A difference-in-difference analysis
Source: PLoS One. 2022 Nov 28;17(11):e0278026. doi: 10.1371/journal.pone.0278026 (PMC9704679; doi:10.1371/journal.pone.0278026)
Supplement: S1 Table — Description of data: The Chinese government issued the National Essential Public Health Services Package (NEPHSP) in 2009; this initiative provides free public health services, including health education, regular health checkups, and regular follow-ups. (DOCX) [file pone.0278026.s005.docx]

| **S1 Table** **Types of services included in NEPHSP in 2009**[1] | |
| --- | --- |
| Types | Details |
| Health records management | Establishing and updating health records and health information for residents who have lived in the area for more than 6 months |
| Health education | Health education and publications about unhealthy lifestyle, risk factors, and diseases to all the residents in the area |
| Health services for children aged 0 to 36 months | Home visits newborn infants, physical examinations for children, and health education and guidance to parents of children aged 0 to36 months |
| Maternal health services | Maternity care before and after delivery, and a post-natal physical examination 42 days after delivery |
| Older people’s health services | Physical examinations, health advice, guidance, and intervention for all those aged over 65 |
| Immunizations | Routine immunizations for children aged 0 to 3 and for vulnerable older people |
| Infectious disease reporting and treatment | Registering, reporting, and managing patients with (suspected) notifiable diseases and their close contacts |
| Health services for patients with hypertension | Establishing health records, screening and following-up and systematic physical examinations of anyone aged over 35 with hypertension |
| Services for patients with type II diabetes | Establishing health records, screening and following-up and systematic physical examinations for anyone aged over 35 with Type II diabetes |
| Services for patients with severe mental illness | Establishing health records and providing follow-up services for any patients with severe mental illness who are living at home |

**References**

[1] Wang Z, Chen Z, Zhang L, Wang X, Hao G, Zhang Z, et al. Status of Hypertension in China: Results From the China Hypertension Survey, 2012-2015. Circulation 2018;137:2344–56. https://doi.org/10.1161/CIRCULATIONAHA.117.032380.
