# Supplementary material for: The impact of increasing expenditure on National Essential Public Health Services on the medical costs of hypertension in China: A difference-in-difference analysis
Source: PLoS One. 2022 Nov 28;17(11):e0278026. doi: 10.1371/journal.pone.0278026 (PMC9704679; doi:10.1371/journal.pone.0278026)
Supplement: S2 Table — Description of data: Comparison of samples data before and after matching, and the groups no significant difference. (DOCX) [file pone.0278026.s006.docx]

| **S2 Table Comparison of samples before and after PSM** | | | | | | |
| --- | --- | --- | --- | --- | --- | --- |
|  | **Before matching** | | **After matching** | | **χ2** | **P** |
| **N** | **3192** |  | 1956 |  |  |  |
| **Age** |  |  |  |  |  |  |
| 45-54 | 869 | 27.22 | 521 | 26.62 | 0.74 | 0.689 |
| 55-64 | 1284 | 40.24 | 811 | 41.45 |  |  |
| ≥65 | 1039 | 32.55 | 625 | 31.94 |  |  |
| **Sex** |  |  |  |  |  |  |
| Men | 1409 | 44.14 | 906 | 46.31 | 2.32 | 0.127 |
| Women | 1783 | 55.86 | 1050 | 53.69 |  |  |
| **Marital** |  |  |  |  |  |  |
| Cohabitant | 2573 | 80.62 | 1616 | 82.61 | 3.23 | 0.072 |
| Single | 619 | 19.38 | 340 | 17.39 |  |  |
| **Work** |  |  |  |  |  |  |
| Not farmer | 752 | 23.55 | 434 | 22.19 | 1.29 | 0.257 |
| Farmer | 2440 | 76.45 | 1522 | 77.81 |  |  |
| **Education** |  |  |  |  |  |  |
| Less than lower secondary education | 2867 | 89.82 | 1792 | 91.62 | 5.11 | 0.078 |
| Upper secondary & vocational training | 275 | 8.62 | 143 | 7.30 |  |  |
| Tertiary education | 50 | 1.57 | 21 | 1.07 |  |  |
| **Household income** |  |  |  |  |  |  |
| Poor | 801 | 25.09 | 461 | 23.55 | 2.19 | 0.534 |
| Low income | 796 | 24.93 | 501 | 25.63 |  |  |
| Middle income | 800 | 25.05 | 482 | 24.62 |  |  |
| High income | 796 | 24.93 | 512 | 26.20 |  |  |
| **Health insurance** |  |  |  |  |  |  |
| No | 207 | 6.49 | 132 | 6.73 | 0.10 | 0.749 |
| Yes | 2968 | 92.98 | 1824 | 93.27 |  |  |
| **Residence** |  |  |  |  |  |  |
| Urban | 1266 | 39.66 | 743 | 38.00 | 1.43 | 0.231 |
| Rural | 1926 | 60.34 | 1213 | 62.00 |  |  |
| **BMI(kg/cm2)** |  |  |  |  |  |  |
| Normal weight (18.5-23) | 907 | 28.41 | 600 | 30.67 | 2.84 | 0.241 |
| Underweight (＜18.5） | 118 | 3.69 | 91 | 4.65 |  |  |
| Overweight or obesity (≥23) | 2167 | 67.90 | 1255 | 64.14 |  |  |
| **Self-reported health status** |  |  |  |  |  |  |
| Fair | 1503 | 47.09 | 909 | 46.46 | 3.92 | 0.27 |
| Poor | 1104 | 34.58 | 716 | 36.58 |  |  |
| Good | 410 | 12.86 | 244 | 12.46 |  |  |
| Very good or Excellent | 175 | 5.48 | 88 | 4.51 |  |  |
